# Multi-omics revealed GOT1/ALDH3A1 pathway attenuated head and neck squamous cell carcinoma and increased cisplatin sensitivity through ROS induced by mitochondrial dysfunction

**DOI:** 10.1080/13510002.2025.2588031

**Published:** 2025-12-01

**Authors:** Zhihui Liu, Baoai Han, Keshu Liu, Peng Zhou, Zehua Lin, Jiawen Li, Weisong Cai, Fangzi Ke, Yifan Hu, Jiahao Meng, Anbang Zhao, Shuang Li, Shuo Huang, Xiong Chen

**Affiliations:** a Department of Otorhinolaryngology-Head and Neck Surgery, Zhongnan Hospital of Wuhan University, Wuhan, People’s Republic of China; b Sleep medicine center, Zhongnan Hospital of Wuhan University, Wuhan, People’s Republic of China

**Keywords:** head and neck squamous cell carcinoma, GOT1, ALDH3A1, mitochondrial dysfunction, ROS, cisplatin, proteomics, metabolomics

## Abstract

Current treatment options for head and neck squamous cell carcinoma (HNSCC) are limited. Aspartate aminotransaminase (GOT1) plays an important role in cancer development but its role in HNSCC remains unknown. We combined proteomics and metabolomics to identify high GOT1expression in human cancer tissues. The effects of GOT1 knockdown on cancer cell proliferation were confirmed using CCK8, wound healing assays, colony formation assays, and EdU assays. The anti-apoptotic ability of cancer cells was evaluated using TUNEL assay and flow cytometry. GOT1 knockdown caused mitochondrial dysfunction and was characterized by reduced mitochondrial membrane potential and altered expression of mitochondrial electron transport chain complexes and key transcription factors, as measured by JC-1 and qRT-PCR. Given that mitochondria are the primary source of reactive oxygen species (ROS), we assessed cellular ROS and mitochondrial superoxide levels by flow cytometry and found a significant increase. GOT1 knockdown increased the sensitivity of cells to cisplatin and decreased the volume of tumors in vivo. In summary, GOT1 knockdown inhibited proliferation and promoted apoptosis via ROS overproduction from mitochondrial dysfunction, thereby increasing cisplatin sensitivity. RNA-seq further identified aldehyde dehydrogenase 3A1 (ALDH3A1) as potentially downstream target of GOT1. These findings suggest that GOT1 knockdown may improve clinical outcomes in HNSCC.

## Background

Head and neck squamous cell carcinoma (HNSCC) is a disease that occurs in the oral cavity, oropharynx, larynx, and hypopharynx, and is closely associated with human papillomavirus infection and tobacco and alcohol abuse[1–3]. As the sixth most common cancer worldwide, nearly one million new cases are diagnosed yearly[4]. The primary treatments for HNSCC are chemoradiotherapy and surgery. Although targeted therapy has made great progress in recent years, the loss of function and detrimental cosmetic outcomes seriously affect the lives of survivors[1].

Cytosolic aspartate aminotransaminase (GOT1) is the core enzyme involved in the reversible synthesis of glutamate and oxaloacetate using alpha-ketoglutarate and aspartate. GOT1 is closely associated with cancer, and its high expression can promote the development of tumors such as pancreatic cancer[5], colorectal cancer[6], and breast cancer[7], or as a potential biomarker such as a diagnostic marker for prostate cancer[8] or a prognostic marker for acute myeloid leukemia[9]. Glucose and glutamate are the major providers of carbon for cell proliferation, with glutamate contributing the most to protein biosynthesis[10]. GOT1 knockdown in a glutamate-deficient environment results in cell death[11]. Adding aspartate can rescue cancer cells with electron transport chain damage from the proliferation inhibition[12], whether GOT1 inhibition directly induces mitochondrial dysfunction remains unclear. Some studies have found that GOT1 also regulates glycolysis, although the specific mechanisms remain unknown.

A low to moderate increase in reactive oxygen species (ROS) is essential in cancer cells to support proliferation, invasion, drug resistance, migration, and angiogenesis[13], whereas high levels of ROS can cause cell death[14]. Disturbing the antioxidant ability of cancer cells is a reasonable anticancer treatment[15,16].

In this study, we used multi-omics to confirm high levels of GOT1 in HNSCC. GOT1 knockdown can disturb redox homeostasis through mitochondrial dysfunction to reverse the outcome of proliferation and anti-apoptosis in HNSCC and increase sensitivity to cisplatin. Aldehyde dehydrogenase 3A1 (ALDH3A1) is a potential downstream target that may contribute to the disruption of glycolysis in cancer cells.

## Methods and materials

### Ethics approval and consent to participate

Human samples were collected from patients diagnosed with HNSCC at the Zhongnan Hospital of Wuhan University. All patients understood the use of the samples and signed a consent form. This study was performed in accordance with the Declaration of Helsinki and was approved by the Ethics Committee of Zhongnan Hospital of Wuhan University (approval number: 2021058).

### Cell culture

Detroit 562(D562) and human laryngeal cancer cell line TU686 (TU686) were purchased from Procell (Wuhan, China). D562 and TU686 cells were cultured in Dulbecco's Modified Eagle Medium (DMEM) and 1640 medium adding 10%(v/v) fetal bovine serum (FBS; HYCEZMBIO, Wuhan, China), respectively. The cell incubator was set at 37°C with 5% CO_2._

### Cell transfection

HEK-293 T cells (human embryonic kidney cells) were purchased from ProCell (Wuhan, China) and cultured in DMEM with 10%(v/v) FBS until 80% confluence was reached. The target plasmid was obtained from Tsingke Biotech (Beijing, China), and the sequences are provided in Supplementary Table S1. Lentiviral packaging was performed by mixing plasmid 1 (vector plasmid, 4 μg), plasmid 2 (packaging plasmid, 4 μg), and the target plasmid (8 μg) in 1 mL DMEM, followed by the addition of 32 μL Polyethylenimine Linear 4 K (Servicebio, Wuhan, China) with gentle mixing. The mixture was incubated at room temperature for 15 min and then added dropwise to 293 T cells, which were then incubated for 48 h. The viral supernatant from 293 T cells was collected and filtered through a 0.45 μm filter. A transduction mixture was prepared at a culture medium: viral supernatant: polybrene ratio of 1:1:1000. The original medium of the D562 and TU686 cells was removed, and the transduction mixture was added dropwise. After 6 h, an equal volume of fresh medium supplemented with 10% (v/v) FBS was added. 48 h post-transduction, the transduction mixture was removed and replaced with fresh medium containing 10% (v/v) FBS. Puromycin (2 μg/mL) was added to the medium for selecting successfully transduced cells. Cell viability was monitored every 12 h, and selection was terminated when all untransfected cells died. Transfection efficiency was assessed using western blotting and qRT-PCR.

### Cell counting kit-8

Cells were seeded in 96 well plates at a density of 1000 cells/well. After overnight incubation, a cell counting kit-8 working solution (HYCEZMBIO, Wuhan, China) was prepared according to the manufacturer’s instructions and added to the medium for seven consecutive days. Incubating at 37°C for 1 h, the OD value at 450 nm was recorded using a VICTOR Nivo™ multimode plate reader (Revvity, Finland).

### Wound healing

Cells were seeded in 6 well plates and incubated until the density reached 90% confluence. The wound areas were scratched using sterile pipette tips and images were captured immediately. Images captured at the same position 2 days later were compared. The percentage of the migration area was analyzed using ImageJ software.

### Colony formation

Cells were seeded at 6 well plates at a density of 500 cells/well. After culturing for 14 days, cells were fixed with 4% (w/v) paraformaldehyde fixed solution, and cell permeability was increased with 0.3% (v/v) Triton for 15 min. After washing twice with Phosphate Buffered Saline (PBS), the cells were stained with crystal violet. The number of colonies was calculated using ImageJ software.

### Edu assay

EdU assay kit was purchased from Service Bio (Wuhan, China). According to the instructions, the EdU working solution was added to the medium and incubated with cells for 2 h. The cells were then washed with PBS and permeabilized with 0.3% (v/v) Triton. Fluorescent dye iF594 was added and incubated for 30 min in the dark. Hoechst 33342 was used to stain the cell nuclei. Four to seven views per sample were captured using an inverted fluorescence microscope (Olympus, Tokyo, Japan). The percentage of EdU-positive cells was evaluated using ImageJ software.

### Flow cytometry

Annexin V-FITC/PI apoptosis kits were provided by MULTISCIENCES (Zhejiang, China). The cell supernatant was collected and centrifuged together with the cells digested with 0.25% (w/v) EDTA trypsin at a cell density of 70% under a microscope. The cell pellet was retained after centrifugation. According to the instructions, cells were resuspended in 1x Binding Buffer (500μL/tube). After adding 5μL Annexin V-FITC and 10μL PI to each tube, the mixture was gently pipetted to mix and incubated at room temperature in the dark for 5 min. A CytoFLEX flow cytometer was used to detect Annexin V-FITC through the FITC channel (Ex/Em = 488 nm/530 nm) and PI through the PI channel (Ex/Em = 535 nm/615 nm), and 10000 cells were counted. After excluding debris and identify doublets, we identified the cell population into three parts: early apoptotic cells: FITC^+^ and PI^-^; late apoptotic cells: FITC^+^ and PI^+^; live cells: FITC^-^ and PI^-^. The results were analyzed using FlowJo software (version 10.8.1).

The Reactive Oxygen Species Detection Kit was purchased from NJJCBIO (Jiangsu, China). The cells were gently washed once with PBS and digested with 0.25% (w/v) EDTA trypsin when the cell density was observed to be 90% under a microscope. The cell pellet was retained after centrifugation. The DCFH-DA stock solution was diluted with PBS to a working solution concentration of 10μM. Cells were resuspended in the working solution and incubated in a 37°C incubator for 30 min, mixing upside down every 5 min. After incubation, the cells were centrifuged to obtain the pellets. The cells were washed twice with PBS and resuspended for detection. A CytoFLEX flow cytometer was used to detect the cell fluorescence intensity through the FITC channel (Ex/Em = 488 nm/530 nm), and 10000 cells were counted. After excluding debris and identifying doublets, fluorescence intensity was used to quantify ROS levels in single cells. The results were analyzed using FlowJo software (version 10.8.1).

The MitoSOX-GREEN Detection Kit was purchased from Thermo Fisher Scientific (Waltham, MA, USA). The cells were gently washed once with PBS and digested with 0.25% (w/v) EDTA trypsin when the cell density was observed to be 90% under a microscope. The cell pellet was retained after centrifugation. According to the instructions, a working solution of MitoSOX-GREEN with a concentration of 1μM was prepared. Cells were resuspended in the working solution and incubated in a 37°C incubator in the dark for 30 min. A CytoFLEX flow cytometer was used to detect the cell fluorescence intensity through the FITC channel (Ex/Em = 488 nm/530 nm), and 10000 cells were counted. After excluding debris and identifying doublets, the fluorescence intensity was used to quantify the level of MitoSOX in single cells. The results were analyzed using FlowJo software (Version: 10.8.1).

### Western blot

RIPA buffer was used to extract proteins from cancer cell lines. After adjusting the concentration of proteins with BCA, SDS-PAGE was performed. Proteins were transferred to a PVDF membrane and blocked with 5% (w/v) skim milk for 1 h. PVDF membranes were incubated with primary antibodies against GAPDH (60004-1-Ig; Proteintech, Wuhan, China), GOT1(A5822; ABclonal, Wuhan, China), and ALDH3A1(A23088; ABclonal, Wuhan, China) overnight at 4 °C on a shaker. The membrane was washed three times with Tris-buffered saline with Tween-20, each for 10 min, followed by incubation with secondary antibodies anti-mouse IgG (RGAM001, Proteintech, Wuhan, China) and anti-rabbit IgG (RGAR001, Proteintech, Wuhan, China) at room temperature on a shaker for 1 h. Finally, the results were detected using an ECL imaging system (BIO-RAD, USA).

### Quantitative real-time PCR

Total RNA was extracted using total RNA solution kits (FOREGENE, Sichuan, China). HiScript IV All-in-One Ultra RT SuperMix for qPCR (Vazyme, Jiangsu, China) was used to obtain cDNA and NanoDrop One/OneC (Thermo Fisher Scientific, USA) was used to determine the concentration. Primers were provided by Tingke Biotech (Beijing, China), and the sequences are summarized in Supplementary Table S2. The 2×Universal Blue SYBR Green qPCR Master Mix (Servicebio, Wuhan, China) was used for qRT–PCR. The results were analyzed using the 2^-△△^Ct method.

### Immunohistochemistry

Immunohistochemistry was performed on formalin-fixed, paraffin-embedded human tissue sections (4 μm thick) to evaluate the protein expression and localization of GOT1, ALDH3A1, Ki67, and proliferating cell nuclear antigen (PCNA). Briefly, the tissue sections were deparaffinized in xylene and rehydrated using a graded ethanol series. Antigen retrieval was performed by heating the sections in a sodium citrate buffer (10 mM, pH 6.0). To quench the endogenous peroxidase activity, the sections were treated with 3% (w/v) hydrogen peroxide in methanol for 20 min at room temperature. After blocking with normal serum to minimize non-specific binding, the sections were incubated overnight at 4 °C in a humidified chamber with the following primary antibodies: anti-GOT1 (A5822, ABclonal, Wuhan, China), anti-ALDH3A1 (A23088, ABclonal, Wuhan, China), anti-Ki67 (A20018, ABclonal, Wuhan, China), and anti-PCNA (A13336, ABclonal, Wuhan, China). After washing, the sections were incubated with the appropriate biotinylated secondary antibody, followed by incubation with a streptavidin-horseradish peroxidase complex. Immunoreactive signals were developed using a 3,3'-diaminobenzidine chromogen substrate, and the sections were counterstained with Mayer's hematoxylin. Finally, sections were dehydrated, cleared in xylene, and mounted with neutral balsam. The stained images were captured using a biological microscope (Olympus CX21FS1). Protein expression was evaluated by two independent pathologists blinded to the sample groups based on the staining intensity and percentage of positive cells.

### Methylation specific PCR

Methylation status of the ALDH3A1 promoter was determined using methylation-specific PCR(MSP). Briefly, DNA was isolated from D562 by using a DNA extraction assay (Servicebio, Wuhan, China) and its concentration was assessed using a NanoDrop One/OneC (Thermo Fisher Scientific, USA). The DNA was treated with a DNA Bisulfite Conversion Kit (Servicebio, Wuhan, China) and manufactured following the instructions to achieve the conservation of DNA. Specific primers were designed (sequences are provided in Supplementary Table S3) and the segments were amplified by PCR. Finally, agarose gel electrophoresis and Ultraviolet light imaging were performed.

### JC-1 assay

A mitochondrial membrane potential assay kit with JC-1 was purchased from Beyotime (Shanghai, China). Experiments were conducted according to the manufacturer’s instructions. Briefly, cells were incubated with JC-1 working solution for 30 min and washed with buffer. The results of inverted fluorescence microscopy and the ratio of JC-1 aggregate to monomers were used to evaluate the degree of mitochondrial membrane damage.

### Subcutaneous tumor model

All procedures were approved and supervised by the Ethics Committee of the Zhongnan Hospital of Wuhan University (approval number: WP20230601). The number of animals was minimized, and pain was controlled. Balb/c-nu mice at 4 weeks years old were purchased from Moulai Bao Co. (Wuhan, China). After acclimatization to a specific pathogen-free bleeding environment, the mice were randomly divided into two groups. The control group was injected with the D562-NC cell suspension, whereas the other group was injected with the D562-Sh1-GOT1 cell suspension. Tumor volumes were recorded 10 days after injection for 5 days at a time. The mice were sacrificed 30 days after injection.

### Untargeted metabolomic sequencing

The metabolites were extracted by adding a precipitator into human tissues, which were then ground, deposited, centrifuged at 25,000** **g for 15** **min at 4 °C, and dissolved to attain the final supernatant. d3-Leucine, 13C9-Phenylalanine, d5-Tryptophan, and 13C3-Progesterone were selected as internal standards. Waters UPLC I-Class Plus (Waters, USA) combined with a Q Exactive high-resolution mass spectrometer (Thermo Fisher Scientific, USA) was used to isolate and detect the metabolites. The LC-MS settings were as follows: BEH C18 column (1.7μm, 2.1 × 100** **mm, Waters, USA); mobile phase for positive ion mode: 0.1% (v/v) formic acid in water (A) and 0.1% (v/v) formic acid in methanol (B); mobile phase for negative ion mode: 10** **mM ammonium formate in water (A) and 10** **mM ammonium formate in 95% (v/v) methanol (B); gradient elution: 0–1** **min, 2% B; 1–9** **min, 2%–98% B; 9–12** **min, 98% B; 12–12.1** **min, 98% B to 2% B; 12.1–15** **min, 2% B; flow rate: 0.35** **mL/min; column temperature: 45 °C; injection volume: 5μL. The MS settings were as follows: mass range, 70–1050** **m/z; resolution, 70000 for MS1 and 17500 for MS2; AGC target, 3 × 10^^6^ for MS1 and 1 × 10^^5^ for MS2; maximum injection time, 100** **ms for MS1 and 50** **ms for MS2; and HCD normalized collision energies, 20, 40, and 60** **eV. Data were collected and analyzed using Compound Discoverer 3.3 (Thermo Fisher Scientific, USA). The metabolites were identified using BGI Metabolome Database (BMDB), mzCloud database, and ChemSpider online database, resulting in a data matrix containing information such as metabolite peak areas and identification results. This data matrix was used for further bioinformatic analyses. The false discovery rate (FDR) was controlled using the Benjamini-Hochberg method with a threshold of 0.05.

### Proteomics sequencing

Human samples were processed according to the detailed protocol for proteomic analysis. Initially, the samples were ground using beads in the presence of 1 × Cocktail of SDS L3, EDTA, and DTT to facilitate protein extraction. Subsequently, cold acetone was used to precipitate the proteins, and the supernatant was discarded after centrifugation at 25000 × g for 15 min at 4 °C. The protein pellets were dissolved in lysis buffer to obtain the final protein solution.

The protein concentration was measured and adjusted using the Bradford method and SDS-PAGE. The proteins were subsequently hydrolyzed using trypsin to obtain peptide fractions. These peptides were labeled with TMT reagents to facilitate quantification in the subsequent steps.

A Shimadzu LC-20AB liquid-phase system was used for the liquid chromatography. The chromatographic elution peak was recorded at 214 nm/min to monitor the separation. Further separation was performed using an Easy-nLC 1200 system (Thermo Fisher Scientific, CA, USA) with high-performance liquid chromatography. Peptides were reconstituted in mobile phase A (2% ACN (v/v) and 0.1% FA (v/v)) and separated using a mobile phase B gradient (80% ACN (v/v) and 0.1% FA (v/v)) on an Easy-nLC 1200 system. The gradient started at 5% B, increased to 44% over 40 min, then to 60% over 5 min, and finally to 100% over 3 min. It was held at 100% B for 7 min before returning to 5% B over 2 min, and equilibrated for 5 min. The separated peptides were ionized using a nanoESI source and analyzed in data-dependent acquisition mode on an Orbitrap Exploris 480 mass spectrometer.

Raw data were used for further bioinformatic analyses. Double-check FDR was applied to ensure the reliability of peptide identification and quantification. During TMT quantification, a stringent 1% FDR filtering standard was used at the peptide spectrum matching level to ensure the credibility of the peptides entering further steps. After assembling the peptides into proteins, a second 1% FDR filter was applied at the protein level to retain only the proteins that passed the dual FDR validation. For peptide quantification, the parameter ‘Quant_number: At least one unique spectra’ was set. Protein coverage, which indicates the proportion of the full-length protein sequence covered by the identified peptides, was used as the core benchmark for sequence recovery, with higher coverage indicating greater protein credibility. The search was performed using *Swiss-Prot* and NR databases.

### RNA sequencing

Total RNA was extracted from the cells using standard methods, and RNA integrity was assessed using an Agilent 2100 Bioanalyzer. mRNA was purified using poly T-attached magnetic beads and fragmented. The first and second strands were synthesized using M-MuLV Reverse Transcriptase and DNA Polymerase I, respectively. The cDNA fragments with lengths between 250-300bp were selected using the AMPure XP system. The cDNA was amplified by PCR and purified to obtain the final product. Sequencing was performed using the Illumina platform to generate raw reads of 150bp in length. HISAT2 (version 2.0.5) was used for alignment and featureCounts (version 1.5.0-p3) was employed for quantification. The reference genome and annotation build used were hg38. Raw data were used for further bioinformatic analysis.

For differential expression analysis, DESeq2 (version 1.20.0) was utilized with the criteria |log_2_(FoldChange)| ≥  1 and padj ≤ 0.05. The Benjamini-Hochberg method was applied to adjust the *p*-value and control the FDR. The specific formula for adjusting the *p*-value is padj = min{1, (m/i) · p_i_}, where m is the total number of hypothesis tests, i is the rank of the *p*-value among all *p*-values, and p_i_ is the current *p*-value. For Functional Enrichment Analysis, a significance threshold of padj < 0.05 was set to identify significantly enriched terms.

### Statistical analysis

All experiments were conducted independently with at least three biological replicates, and data were analyzed using Excel (Microsoft, WA, USA) and GraphPad Prism (GraphPad Software, CA, USA). Results are presented as the mean ± Standard Error of the Mean (SEM). For comparisons between two independent groups, we initially performed an F-test to assess the homogeneity of variance, followed by a two-tailed Student's t-test. For comparisons involving more than two groups, we employed a one-way Analysis of Variance after confirming homoscedasticity using the Brown-Forsythe test. Additionally, Šídák's multiple comparison test was conducted for further post hoc analyses. Statistical significance was set at a *p*-value less than 0.05.

## Results

### Metabolomics and proteomics revealed GOT1 is overexpressed in HNSCC

**Figure 1.**
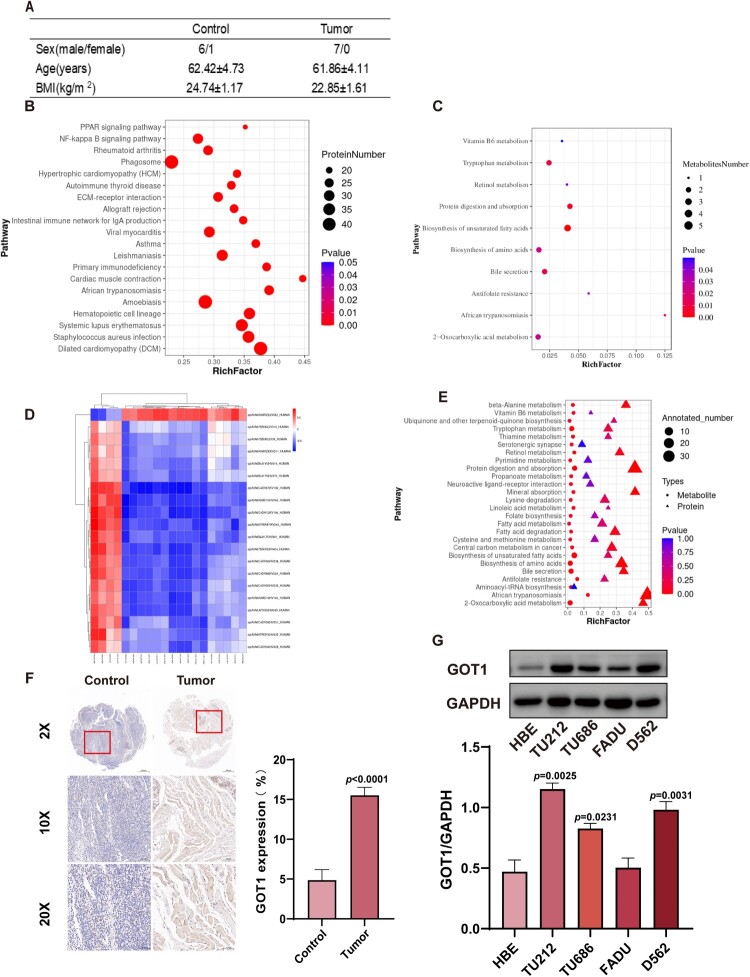
Proteomic and metabolomic analyses revealed a high expression of GOT1 in HNSCC (A) Basic information of patients donating tissues; (B) KEGG enrichment results of the proteomic analysis; (C) KEGG enrichment results of the metabolomic analysis; (D) Heatmap of the top 20 genes using joint analysis of the multi-omics data; (E) KEGG enrichment results of the joint analysis of the multi-omics data; (F) Representative GOT1 staining immunochemical images of human samples and quantitative analysis results; (G) Protein expression of GOT1 in different cell lines (N = 3). GOT1: aspartate aminotransaminase; HNSCC: head and neck squamous cell carcinoma; GAPDH: glyceraldehyde-3-phosphate dehydrogenase; HBE: human bronchial epithelial cells; TU212: human laryngeal cancer cells TU212; TU686: human laryngeal cancer cells TU686; FADU: human pharyngeal squamous cell carcinoma cells; D562: Detroit 562. The data are displayed as mean ± SEM.

Clinical tissue samples were collected from patients with HNSCC who underwent surgery for proteomic analysis. Four samples from each group were selected for the metabolomic analysis. The average age was 62 years in both groups, and the average BMI were 25 and 23, respectively, with no significant difference (Figure 1A). A total of 798 different expression proteins were detected (Figure S1 A), and KEGG enrichment pathways based on these proteins were performed (Figure 1B). LC-MS/MS was used for metabolite detection, and information regarding the evaluation of the effectiveness of the data is provided in the Supplementary Material (supplementary Figure 1C-D). Different metabolites in the two groups were used for further KEGG enrichment analysis (Figure 1C). A combination analysis of proteomics and metabolomics has been used to explore data in-depth[17–20]. The Spearman correlation coefficients of the top 20 differential proteins and differential metabolites are presented in a heatmap (Figure 1D), and all significantly changed proteins and metabolites between the two groups were used for enrichment analysis (Figure 1E).

Compared to normal cells, cancer cells have higher energy requirements and a stronger antioxidant defense system, and inhibiting these functions may be beneficial for tumor treatment[21,22]. Therefore, we selected the cysteine and methionine metabolic pathways, which play important roles in antioxidant activity and energy production, based on the results of combined enrichment analysis for further research, and identified GOT1, whose expression was significantly upregulated in this pathway, as the target for subsequent studies (Figure 1E)[23].

The immunohistochemical results of the tissue microarray demonstrated high expression of GOT1 in cancer tissues (Figure 1F). The expression of GOT1 in the four cancer cell lines was tested by WB, and TU686 and D562 were selected for subsequent experiments because of their high expression of GOT1(Figure 1G).

### Knock-down GOT1 inhibited the proliferation and induced apoptosis of HNSCC

**Figure 2.**
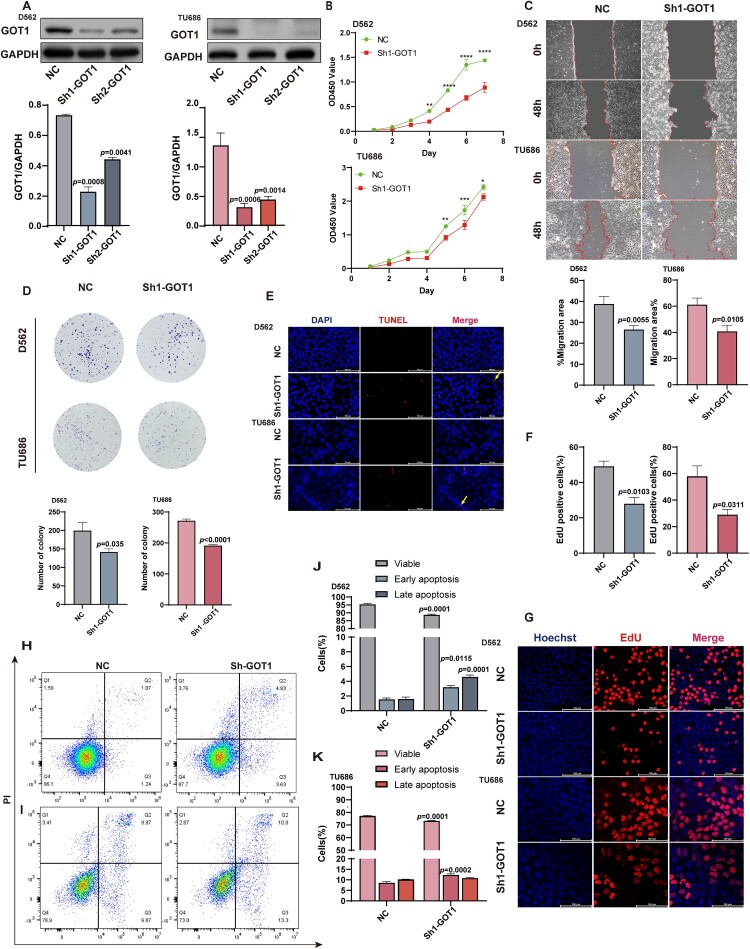
Knocking GOT1 inhibited the proliferation of and promoted apoptosis of cancer cells. (A) Western blotting of GOT1 expression in D562 and TU686 following ShRNA transfection (N = 3); (B) CCK-8 assay for 7 consecutive days demonstrates the effect of D562 and TU686 on cell growth after silencing GOT1 (N = 3); (C) Representative images of wound healing assay and the quantitative analysis results demonstrate the effect of D562 and TU686 on cell migration after silencing GOT1 (N = 6); (D) Representative images of colony formation assay and the quantitative analysis results demonstrate the effect of D562 and TU686 on cell colony-forming ability after silencing GOT1 (N = 6); (E) Representative images of TUNEL staining showing the effect of D562 and TU686 on cell death after silencing GOT1; (F-G) Representative images of the EdU assay and quantitative analysis results showed the effect of D562 and TU686 on proliferation after silencing GOT1 (N = 3); (H-K) Flow cytometry images and quantitative analysis results showing the effect of D562 (H, J) and TU686 (I, K) on anti-apoptosis ability after silencing GOT1 (N = 3). GOT1: aspartate aminotransaminase; TU686: human laryngeal cancer cells TU686; D562: Detroit 562. The data are displayed as mean ± SEM.

To identify the potential effects of GOT1 on HNSCC, stable GOT1 knockdown cell lines were constructed. The knockdown effectiveness was measured by WB (Figure 2A) and qRT-PCR (Figure S2), and sequence number one was selected for further research. The CCK-8 assay results showed that the viability of NC was higher than that of Sh1-GOT1 both in D562 and TU686 cells (Figure 2B). We further compared the migratory and proliferative abilities between the two groups using wound healing assays, and found that GOT1 knockdown significantly reduced the wound closure area and the number of Ki67-positive cells after 48 h. (Figure 2C, Figure S3). A colony formation assay was performed to measure colony forming ability, and similar results were obtained (Figure 2D).

Representative TUNEL staining images indicated the presence of more DNA segments in the Sh1-GOT1 group (Figure 2E). EdU, a thymidine analog, is incorporated into newly synthesized DNA during S phase, and the percentage of EdU-positive cells reflects the proliferative rate. As shown in Figure 2F-G, the nuclei were stained blue, and EdU-positive signals appeared red. Compared to the NC group, the Sh1-GOT1 group exhibited a marked reduction in the percentage of EdU-positive cells, indicating that GOT1 knockdown slowed cancer cell proliferation. Flow cytometry was used to assess survival and apoptosis rates in the two groups. Compared with the control group, the percentage of early and late apoptosis was increased in the Sh1-GOT1 group of D562 cells (Figure 2H, 2J), and only the percentage of early apoptosis was significantly higher than that in the Sh1-GOT1 group in TU686 cells; this pattern was recurrent in later results (Figure 2I, K).

### Knock-down GOT1 induced mitochondria dysfunction and increased the ROS accumulation in cancer cells

**Figure 3.**
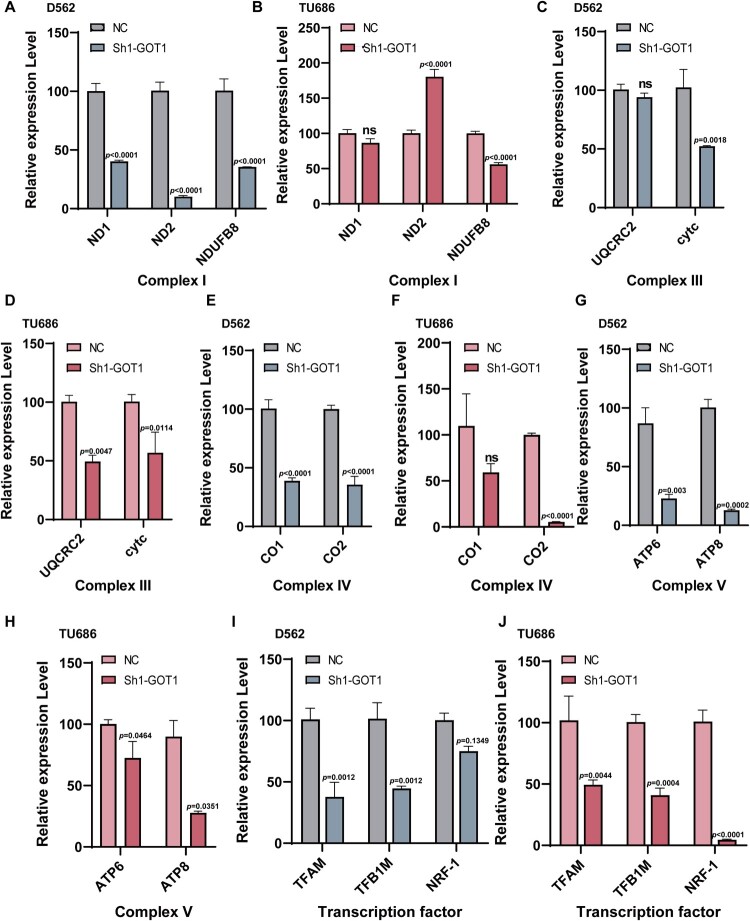
Knocking GOTI resulted in mitochondrial dysfunction in cancer cells. (A-B) Relative mRNA expression of complex I (ND1, ND2, and NDUFB8) of electron transport chain in D562 (A) and TU686 (B) (N = 3); (C-D) Relative mRNA expression of complex III (UQCRC2 and cytc) of electron transport chain in D562 (C) and TU686 (D) (N = 3); (E-F) Relative mRNA expression of complex IV (CO1 and CO2) of electron transport chain in D562 (E) and TU686 (F) (N = 3); (G-H) Relative mRNA expression of complex V (ATP6 and ATP8) of electron transport chain in D562 (G) and TU686 (H) (N = 3); (I-J) Relative mRNA expression of transcription factors (TFAM, TFB1M, and NRF-1) of mitochondria in D562 (I) and TU686 (J) (N = 3). TU686: human laryngeal cancer cells TU686; D562: Detroit 562; ATP6: ATP synthase 6; ATP8: ATP synthase 8; CO1: cytochrome c oxidase subunit I; CO2: cytochrome c oxidase subunit II; cytc: cytochrome C; ND1: NADH dehydrogenase subunit 1; ND2: NADH dehydrogenase subunit 2; NDUFB8: NADH ubiquinone oxidoreductase subunit B8; NRF-1: nuclear respiratory factor 1; TFAM: transcription factor A; TFB1M: transcription factor B1; UQCRC2: ubiquinol-cytochrome c reductase core protein 2. The data are displayed as mean ± SEM.

Previous studies have demonstrated that GOT1 is important for maintaining the redox balance of mitochondria through the malate shuttle, and inhibiting GOT1 can reduce the basal oxygen consumption rate to potentiate ferroptosis in pancreatic cancer cells[24–26]. Therefore, we hypothesized that GOT1 knockdown could damage mitochondrial function and disrupts the redox balance. First, we measured the mRNA expression levels of genes related to the electron transport chain. Almost all genes decreased significantly compared to the NC group, except for ND1 and CO1 in TU686 cells and UQCRC2 in D562 cells (Figure 3A-H). Notably, ND2 expression was higher in the Sh1-GOT1 group in TU686. ND2 is a subunit of NADH that is highly expressed in colorectal cancer[27]. However, the reason for the high expression of ND2 in TU686 cells requires further research.

TFAM and TFB1M are the core transcription factors that activate mtDNA transcription and repair. NRF-1 is responsible for redox regulation, and decreased NRF-1 levels lead to severe oxidative stress[28]. They all decreased significantly after GOT1 knockdown in D562 and TU686 cells. The results indicated that severe mitochondrial damage was induced by GOT1 knockdown in HNSCC (Figure 3I-J).

**Figure 4.**
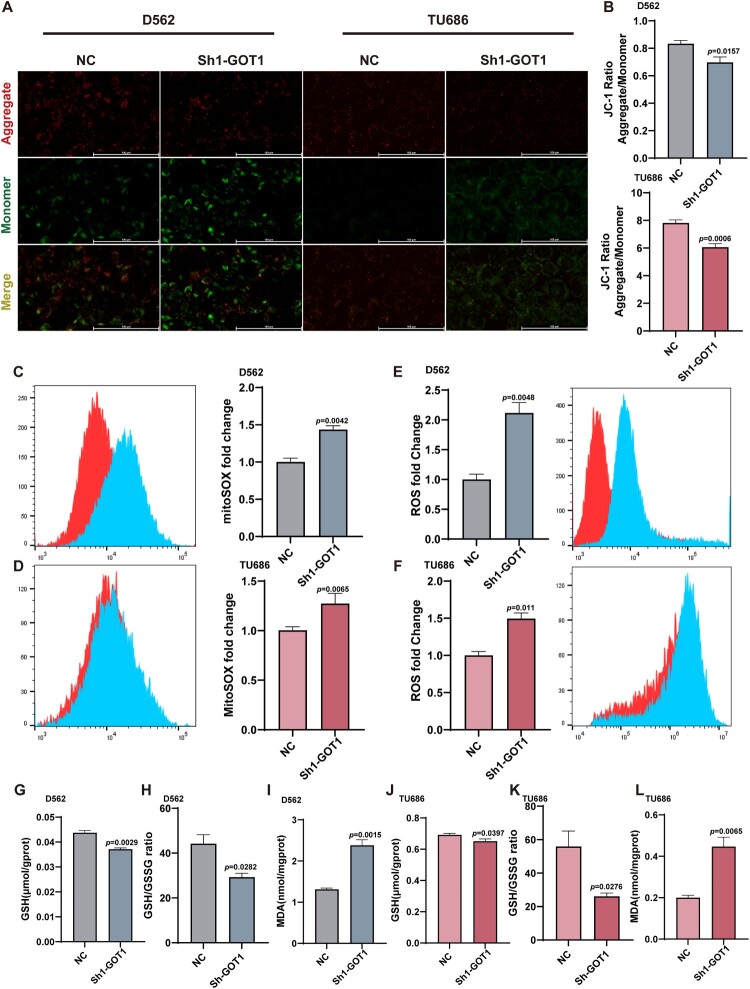
Knocking GOTI resulted in the overproduction of ROS in cancer cells. (A-B) Mitochondrial membrane potential tested by JC-1 and (A) the ratio of the JC-1 aggregate to monomer (B) (N = 3); (C-D) Relative mitoSOX level tested using flow cytometry and quantitative analysis results of D562 (C) and TU686 (D) (N = 3); (E-F) Relative ROS level tested using flow cytometry and quantitative analysis results of D562 (E) and TU686 (F) (N = 3); (G-I) Level of GSH, GSH/GSSG ratio and MDA in D562 (N = 3); (J-L) Level of GSH, GSH/GSSG ratio and MDA in TU686 (N = 3). TU686: human laryngeal cancer cells TU686; D562: Detroit 562; ROS: reactive oxygen species; mitoSOX: mitochondrial superoxide indicator; MDA: malondialdehyde, GSH: glutathione; GSSG: glutathione disulfide. The data are displayed as mean ± SEM.

Mitochondrial membrane potential (ΔΨm) is essential for normal mitochondrial function. JC-1 aggregates emit red fluorescence, indicating intact ΔΨm, whereas JC-1 monomers emit green fluorescence, signifying a decrease in ΔΨm. The JC-1 aggregate/monomer ratio was significantly lower in the Sh1-GOT1 group than that in the NC group, indicating that GOT1 knockdown caused severe mitochondrial damage in HNSCC cells (Figure 4A-B). We further used flow cytometry to detect the superoxide anion radicals in the cell and mitochondria and confirmed that oxidative stress was more severe in the Sh1-GOT1 group (Figure 4C-F). Glutathione (GSH) is an important intracellular antioxidant that exists in cells in either its form (GSH) or oxidized form (glutathione disulfide, GSSG). Maintaining a normal GSH/GSSG ratio is crucial for cell survival[29]. Malondialdehyde (MDA) is commonly used as a marker of lipid peroxidation. The decrease in GSH, GSH/GSSG ration, and increase in MDA levels suggested that the antioxidant capacity was weakened in the Sh1-GOT1 group (Figure 4G-L).

We then employed N-acetylcysteine (NAC), a well-characterized ROS inhibitor[30], to further confirm the critical role of excessive ROS production in the therapeutic effects induced by GOT1 knockdown. The results showed that NAC treatment reversed the changes in Sh1-GOT1 group in terms of cell growth and migration ability, accompanied by an increased proportion of Ki67-positive cells (Figure S4).

### ALDH3A1 was the potential downstream of GOT1

**Figure 5.**
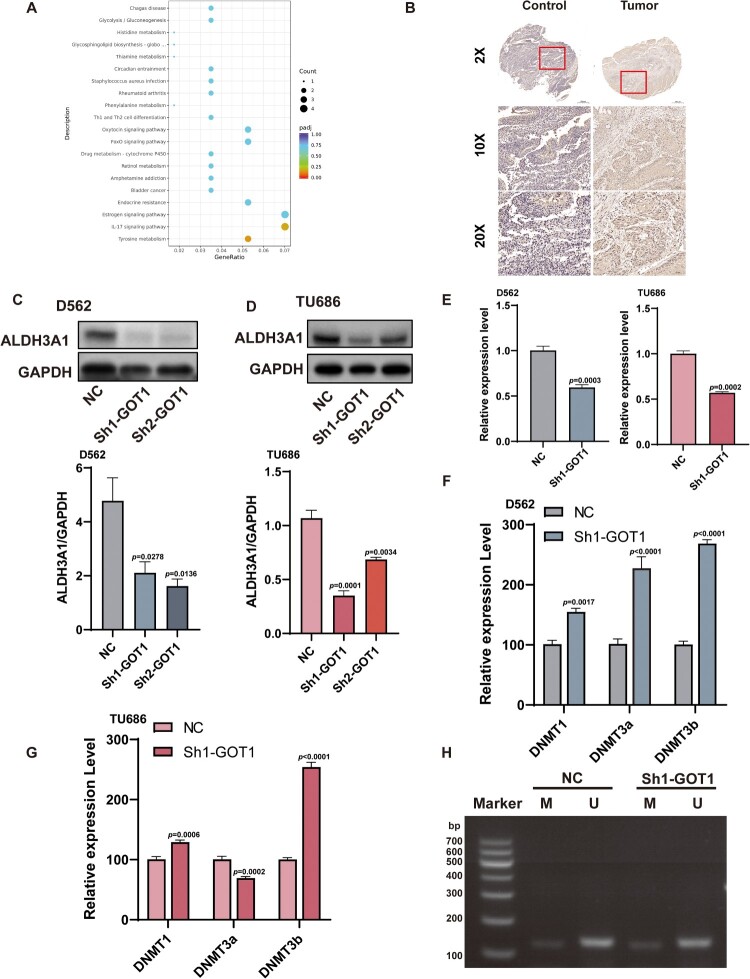
ALDH3A1 was the downstream target of GOT1. (A) RNA-seq-based KEGG pathway enrichment analysis of differentially expressed genes in D562 after GOT1 knockdown; (B) Protein expression of ALDH3A1 in human tissues; (C-D) Protein expression of ALDH3A1 in cancer cell lines after knocking GOT1 (N = 3); (E) Relative mRNA expression of ALDH3A1 in cancer cell lines after knocking GOT1 (N = 3); (F-G) Relative mRNA expression of methylating enzymes (DNMT1, DNMT3a, and DNMT3b) in D562 and TU686 (N = 3); (E) Results of MSP in D562. ALDH3A1: aldehyde dehydrogenase 3A1; DNMT1: DNA methyltransferase 1; DNMT3a: DNA methyltransferase 3 alpha; DNMT3b: DNA methyltransferase 3 beta, MSP: methylation-specific PCR; TU686: human laryngeal cancer cells TU686; D562: Detroit 562. The data are displayed as mean ± SEM.

RNA-seq was used to explore potential downstream targets of GOT1. A stable knockdown GOT1 cell line was established using D562 cells via cell transfection, and a KEGG spot image of the differentially expressed genes is presented (Figure 5A). The tyrosine metabolism pathway showed the most significant differences between the two groups and the expression of ALDH3A1 dropped significantly in that way. The immunohistochemistry results of human samples indicated that ALDH3A1 expression was higher in cancer tissues than in normal tissues (Figure 5B). We found that the mRNA and protein levels of ALDH3A1 decreased after GOT1 knockdown in cancer cells, which was consistent with the sequencing results (Figure 5C-E). Previous studies have demonstrated that secondary metabolites of GOT1 are related to epigenetic mutations. Therefore, we speculate that hypermethylation occurs in the promoter of ALDH3A1. We first assessed the mRNA expression of the classic methylating enzymes DNMT1, DNMT3a, and DNMT3b. All enzymes showed higher expression in the Sh1-GOT1 group, except for DNMT3a in TU686 cells (Figure 5F-G). MSP was performed to determine the methylation status of the ALDH3A1 promoter. However, both groups showed partial methylation and did not show any visual changes after GOT1 knockdown in D562 cells (Figure 5H).

### Knock-down GOT1 increased the sensitivity of cisplatin in cancer cells and limited the growth of tumor *in vivo*

**Figure 6.**
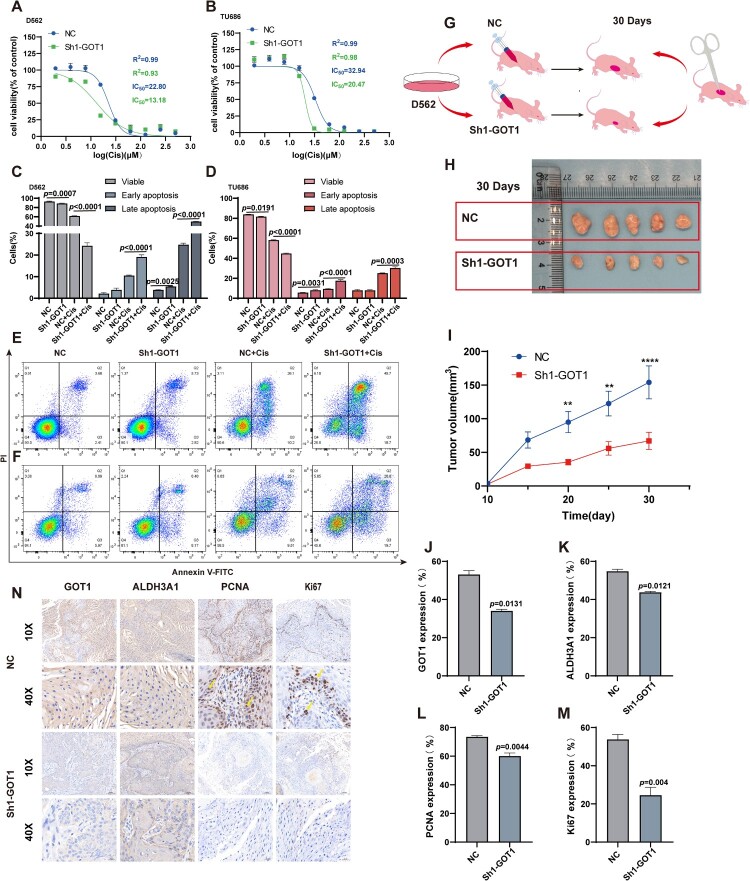
Increased sensitivity of cisplatin in vitro and efficiency of limiting tumor growth in vivo after knocking GOT1. (A) Value of IC_50_ in D562 after treatment of different concentration of cisplatin for 24 h (N = 3); (B) Value of IC_50_ in TU686 after treatment of different concentration of cisplatin for 24 h (N = 3); (C-D) Percentage of cells in different phases of the cell cycle after pretreating with cisplatin for 24 h in D562 and TU686 (N = 3); (E-F) Representative images of flow cytometry of cells pretreated with cisplatin for 24 h in D562 and TU686; (G) Schematic diagram of the animal experiment; (H) Images of dissected tumor tissues (N = 5); (I) Growth curve of the tumors (N = 5); (J-M) Quantitative immunohistochemical results of GOT1 (J), ALDH3A1 (K), PCNA (L) and Ki67 (M) (N = 5); (N) Representative images of immunocytochemistry. ALDH3A1: aldehyde dehydrogenase 3A1; GOT1: aspartate aminotransaminase; PCNA: proliferating cell nuclear antigen; Cis: cisplatin. The data are displayed as mean ± SEM.

Cisplatin is widely used for the treatment of HNSCC. We evaluated the effect of GOT1 knockdown on cisplatin sensitivity. Silencing GOT1 resulted in a 1.7-fold and 1.6-fold decrease in the IC₅₀ of cisplatin in D562 (22.8 μM in NC vs. 13.18 μM in Sh1-GOT1) and TU686 (32.94 μM in NC vs. 20.47 μM in Sh1-GOT1) cells, respectively (Figure 6A–B). Accordingly, 25 and 35 μM cisplatin were selected for subsequent experiments in D562 and TU686 cells, respectively – slightly above the higher IC₅₀ values to ensure measurable cytotoxic effects in both groups[31]. Cell viability and apoptosis were further assessed by flow cytometry after 24** **h of cisplatin pretreatment. The proportion of viable cells markedly decreased following GOT1 knockdown, and this reduction became more pronounced upon cisplatin exposure. Consistently, the percentage of late apoptotic cells was significantly higher in the Sh1-GOT1 + Cis group compared with the NC + Cis group (Figure 6C–F).

Balb/c-nu mice were injected with the cell suspension and sacrificed after one month (Figure 6G). Significant differences in tumor volume were observed in ex vivo photos and were analyzed using weekly data (Figure 6H-I). Immunohistochemistry confirmed that the lower expression of GOT1 and ALDH3A1 was consistent with the smaller tumor volume in the Sh1-GOT1 group. Ki67 is a nuclear protein whose high expression is closely associated with the aggressive potential of tumor cells, making it a widely used marker for assessing proliferation in various cancers, such as pancreatic cancer. Proliferating cell nuclear antigen (PCNA) regulates DNA replication and repair by accurately ensuring nucleic acid metabolism, and its elevated expression reflects active cellular proliferation[32]. Immunohistochemistry staining showed that the expression levels of both markers were lower in the Sh1-GOT1 group than in the NC group (Figure 6J-N).

## Discussion

Cancer is the second leading cause of death in the United States of America. Head and neck cancer accounts for almost 3% of all cancer cases and > 1.5% of all cancer-related deaths[33]. The prognosis of head and neck cancer is poor, with a 50% chance of recurrence and 51% total morality[4]. The limitations of treatment and unsatisfactory treatment outcomes indicate that more precise and effective therapies are required.

Mitochondria produce ATP through oxidative phosphorylation (OXPHOS). Only 1-2% consumed oxygen during this process is utilized for ROS production under normal status and then plays an essential role in some physiological processes, such as the regulation of cellular differentiation and remodeling of chromatin structure[34,35]. Abnormal ROS production is indicative of mitochondrial dysfunction and associated with apoptosis[36]. Previous studies have revealed that complexes I and III of the electron transfer chain are the main source of ROS[37]. Our results show that the mRNA expression of genes related to the electron transfer chain (including complexes I, III, IV, and V) decreased significantly in both D562 and TU686 cells. The transcription factors associated with mitochondrial function exhibited similar patterns. These changes were consistent with the accumulation of ROS in mitochondria and whole cells, as observed by flow cytometry. These results confirmed that GOT1 knockdown can accelerate the apoptosis of cancer cells by damaging mitochondria to increase ROS levels.

Cisplatin is recognized as the reference drug for the treatment of patients with locoregionally advanced HNSCC, but its adverse effects and resistance greatly limit its use[38]. Clinical trials using a combination of cisplatin and radiation have not presented satisfactory results, suggesting that a more effective method to improve the sensitivity of cisplatin and expand the number of eligible patients is urgently needed. This study provides evidence that the IC_50_ and apoptosis rate of cancer cells after treatment with cisplatin for 24 h dramatically decreased and increased, respectively, in the Sh1-GOT1 group. GOT1 inhibitors may function as adjuvant drugs to benefit patients.

Metabolic reprogramming is essential for the development, invasion, and migration of cancer cells. Compared to normal cells, over 50% of ATP is produced by glycolysis in cancer cells, and this preference persists in the presence of oxygen (Warburg Effect)[39]. To meet the need for rapid growth, cancer cells can switch between glycolysis and OXPHOS; inhibiting these processes is a promising method for treating cancer[40,41]. A previous study reported that the combined use of intermittent fasting and metformin could protect against cancer by inhibiting glycolysis and OXPHOS[42]. ALDH3A1 is a member of the aldehyde dehydrogenase family that contributes to the oxidation of aldehydes to carboxylic acids[43]. Previous studies have confirmed that ALDH3A1 can enhance glycolysis in non-small-cell lung cancer[44]. Our study found that ALDH3A1 expression was downregulated following GOT1 knockdown. To confirm the specific role of GOT1 in this regulation, we measured the mRNA expression of DNA methyltransferases, including DNMT1, DNMT3a, and DNMT3b. All DNA methyltransferases showed a significant increase in the Sh1-GOT1 group with DNMT3a in TU686, while the MSP results indicated that there was no visual difference in the methylation of the promoter. Xu et al. found that increasing levels of 2-hydroxyglutarate produced by GOT1 cause differentiation of TH17 cells through hypermethylation of the FOXP3 gene locus[45]. However, W. Xu and his colleague reported a different result that GOT1 promotes the expression of FOXP3[46]. This suggests that the precise function of GOT1 requires further investigation.

## Conclusion

In summary, our study demonstrated that down-regulating the expression of GOT1 can inhibit proliferation, accelerate apoptosis of cancer cell lines, and increase cisplatin sensitivity. The overproduction and accumulation of ROS played an important role in this process, which is caused by vital mitochondrial dysfunction. ALDH3A1 is a potential downstream target of GOT1, as identified by RNA-Seq. Targeting GOT1 is a potential therapeutic strategy for the treatment of HNSCC.

## Supplementary Material

Additional file 1.pdf

Additional file 2.pdf

## Data Availability

The data that support the findings of this study are available from the corresponding author XC, upon reasonable request.
