# Supplementary material for: Multi-omics revealed GOT1/ALDH3A1 pathway attenuated head and neck squamous cell carcinoma and increased cisplatin sensitivity through ROS induced by mitochondrial dysfunction
Source: Redox Rep. 2025 Dec 1;30(1):2588031. doi: 10.1080/13510002.2025.2588031 (PMC12671062; doi:10.1080/13510002.2025.2588031)
Supplement: Additional file 1.pdf [file YRER_A_2588031_SM4256.pdf]

Multi-omics revealed GOT1/ALDH3A1 pathway attenuated head and neck squamous cell carcinoma and increased cisplatin sensitivity through ROS induced by mitochondrial dysfunction

Zhihui Liu[a,b #], Baoai Han[a,b #], Keshu Liu[a,b #], Peng Zhou[a,b#], Zehua Lin[a,b], Jiawen Li[a,b], Weisong Cai[a,b], Fangzi Ke[a,b], Yifan Hu[a,b], Jiahao Meng[a,b], Anbang Zhao[a,b], Shuang Li[a,b *], Shuo Huang[a,b*], Xiong Chen[a,b*]

[a]Department of Otorhinolaryngology-Head and Neck Surgery, Zhongnan Hospital of Wuhan University, Wuhan, 430071, China.

[b]Sleep medicine center, Zhongnan Hospital of Wuhan University, Wuhan,430071, China.

[#] These authors contributed equally to this work

*Corresponding author:

Xiong Chen Email: zn_chenxiong@whu.edu.cn; Tel: +86-13995599373;

ORCID: https://orcid.org/0009-0005-3311-2783;

Shuo Huang Email: clarkehs@whu.edu.cn; Tel: +86-13554492520;

Shuang Li Email: entlishuang@whu.edu.cn; Tel: +86-15623052998;

Address: Zhongnan Hospital of Wuhan University, No.169 Donghu Road, Wuchang District, Wuhan, 430071, China.

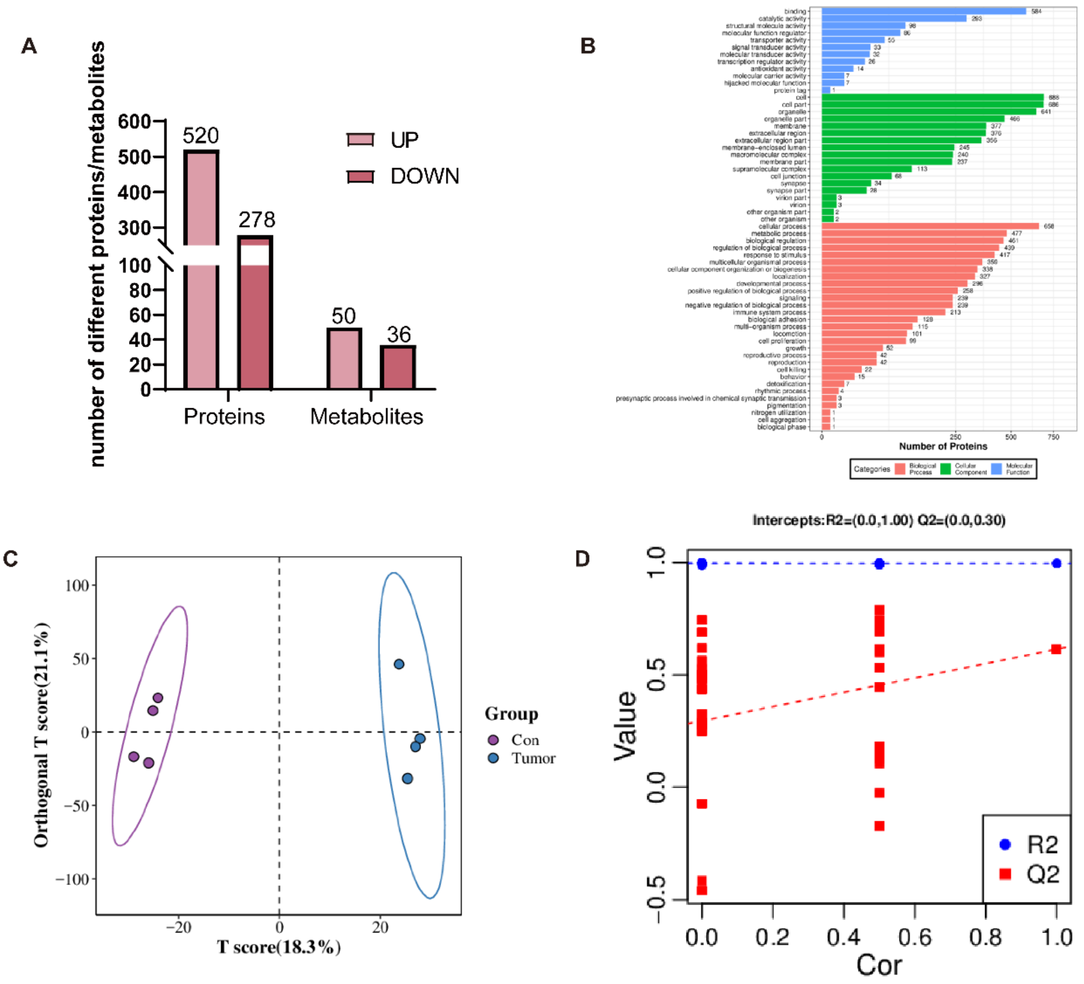

Figure.S1 Bioinformatic analysis of proteomic and metabolomic. (A)The up and down-regulated number of proteins analyzed by proteomic and metabolomic; (B)The GO enrichment analysis of the different expression proteins identified by proteomic; (C)The OPLS-DA image of the sample used for metabolomic; (D)The R2 and Q2 for OPLS-DA.

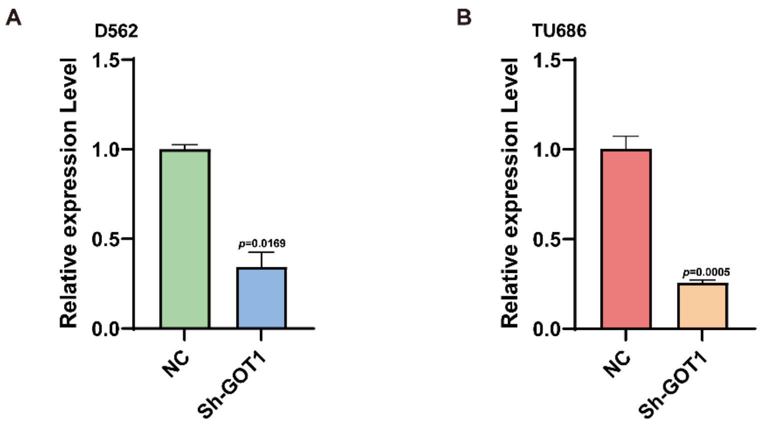

Figure.S2 The mRNA expression of GOT1 in D562 and TU686 after transfection. (A) The mRNA expression of GOT1 in D562 (N=3); (B) The mRNA expression of GOT1 in TU686 (N=3). Mean ± S.E.M.

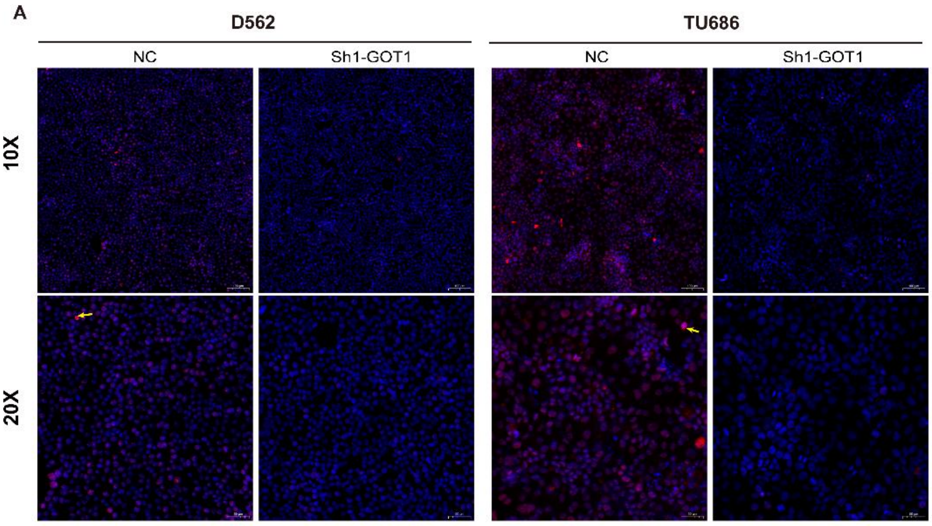

Figure.S3 Immunofluorescence staining of Ki67 in D564 and TU686 48 h after wound healing assay. Yellow arrows in the figure indicate representative Ki67-positive cells.

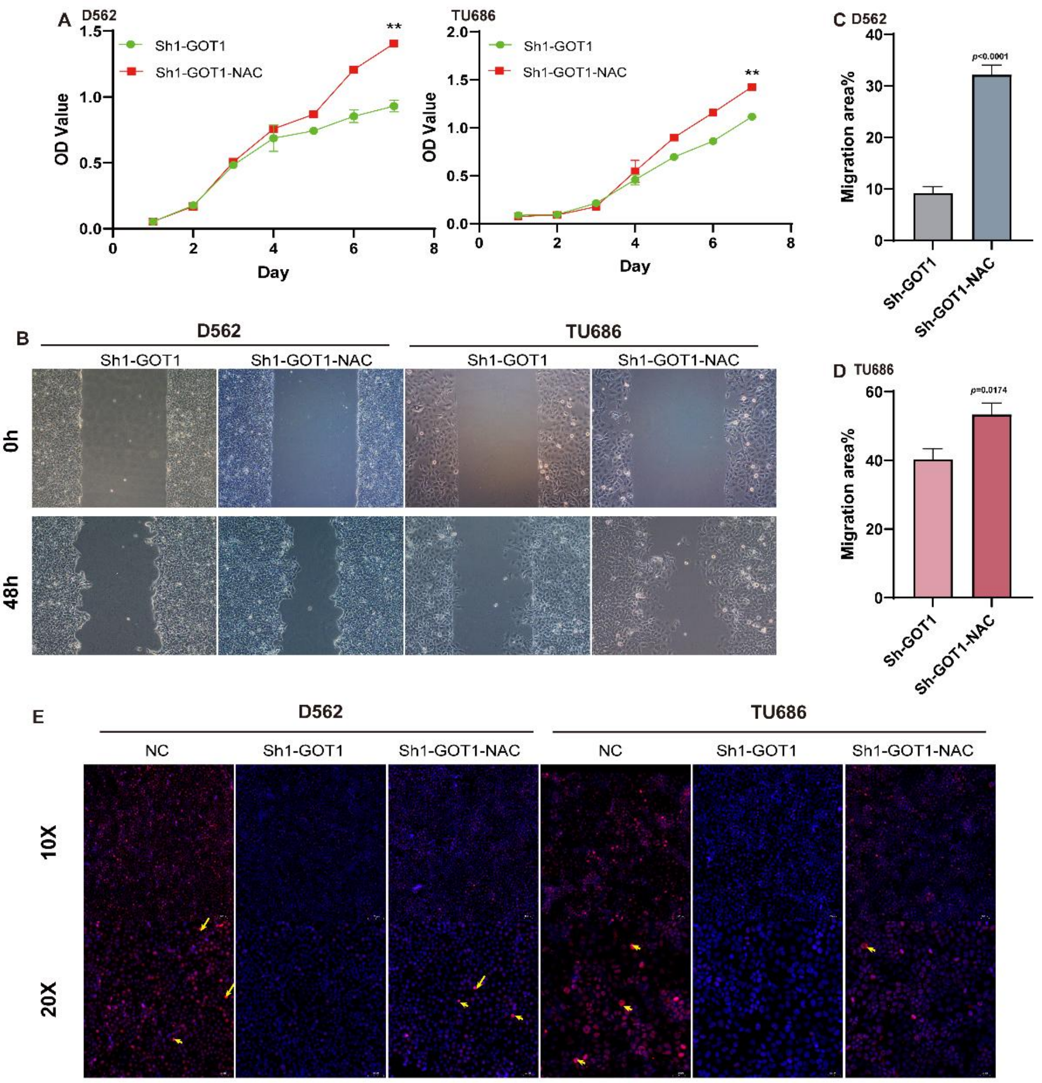

Figure.S4. Mitigation of ROS production abrogated the therapeutic effects induced by GOT1 knockdown in cancer cells. (A) CCK-8 assay for 7 consecutive days showed that cell growth increased in D562 and TU686 cells treated with NAC following GOT1 silencing (N=3); (B-D) Representative images of wound healing assay and the quantitative analysis results showed that cell migration increased in D562 and TU686 cells treated with NAC following GOT1 silencing (N=6); (E) Immunofluorescence staining of Ki67 in D564 and TU686 cells treated with NAC, 48 h after the wound healing assay.

GOT1: aspartate aminotransaminase; TU686: human laryngeal cancer cells TU686; D562: Detroit 562. NAC: N-acetylcysteine, a ROS scavenger. Yellow arrows in the figure indicate representative Ki67-positive cells. The data are displayed as mean ± SEM.

Table S1. The primer sequences used in cell transfection

Gene	Forward/Reverse primer
NC	GAAAGGCTGTTGGCATCAATA
Sh1-GOT1	GCGTTGGTACAATGGAACAAA
Sh2-GOT1	GCTAATGACAATAGCCTAAAT

Table S2. Primer sequences of human genes used for qRT-PCR.

Gene	Forward primer（5'-3'）	Reverse primer（5'-3'）	Accession Code
GOT1	CTGGGAGTGGGAGCATAT	CAAGGGCAAGACGAGAAG	NM_002079.3
ALDH3A1	GTGGGAATGTGAGCTGGAAC	TGTTCTCCAGCAACGACAAGG	NM_000691.5
NDUFB8	CCGCCAAGAAGTATAATATGCGT	TATCCACACGGTTCCTGTTGT	NM_005004.4
UQCRC2	TTCAGCAATTTAGGAACCACCC	GGTCACACTTAATTTGCCACCAA	NM_003366.4
Cytc	CTTTGGGCGGAAGACAGGTC	TTATTGGCGGCTGTGTAAGAG	NM_018947.6
CO1	ACGTTGTAGCCCACTTCCAC	TGGCGTAGGTTTGGTCTAGG	NM_001246910.2
CO2	AATCGAGTAGTACTCCCGATTG	TTCTAGGACGATGGGCATGAAA	NM_000063.6
TFAM	ATGGCGTTTCTCCGAAGCAT	TCCGCCCTATAAGCATCTTGA	NM_001270782.2
TFB1M	AGAGACTTGCAGCCAATACAGG	GTGTCGAACATTGCAGAGGTA	NM_001350501.2
NRF-1	GCTGATGAAGACTCGCCTTCT	TACATGAGGCCGTTTCCGTTT	NM_001040110.2
DNMT1	CCTAGCCCCAGGATTACAAGG	ACTCATCCGATTTGGCTCTTTC	NM_001318731.2
DNMT3a	CCGATGCTGGGGACAAGAAT	CCCGTCATCCACCAAGACAC	NM_001320893.1
DNMT3b	CCCAGCTCTTACCTTACCATCG	GGTCCCCTATTCCAAACTCCT	NM_001424354.1

Table S3. Primer sequences of the promotor of ALDH3A1 used for MSP.

Gene	Forward primer	Reverse primer
ALDH3A1(M)	GTTATGAGTAAGATTAGCGAG GTCG	CTCCTAAATCAAACGCTACAAC G
ALDH3A1(U)	GGTGTTATGAGTAAGATTAGT GAGGTTG	CCTACTCCTAAATCAAACACTA CAACA
