# Supplementary material for: Multi-omics revealed GOT1/ALDH3A1 pathway attenuated head and neck squamous cell carcinoma and increased cisplatin sensitivity through ROS induced by mitochondrial dysfunction
Source: Redox Rep. 2025 Dec 1;30(1):2588031. doi: 10.1080/13510002.2025.2588031 (PMC12671062; doi:10.1080/13510002.2025.2588031)
Supplement: Additional file 2.pdf [file YRER_A_2588031_SM4255.pdf]

Original western blot for Figure 1G

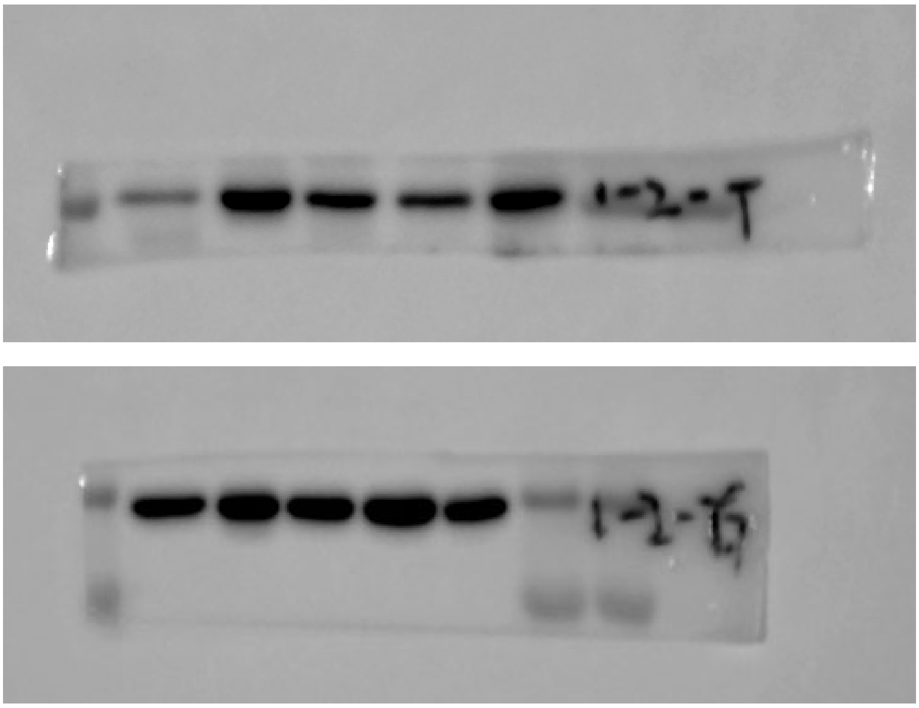

GOT1

GAPDH

Marker HBE TU212 TU686 FADU D562 Marker Marker

Original western blot for Figure 2A

D562

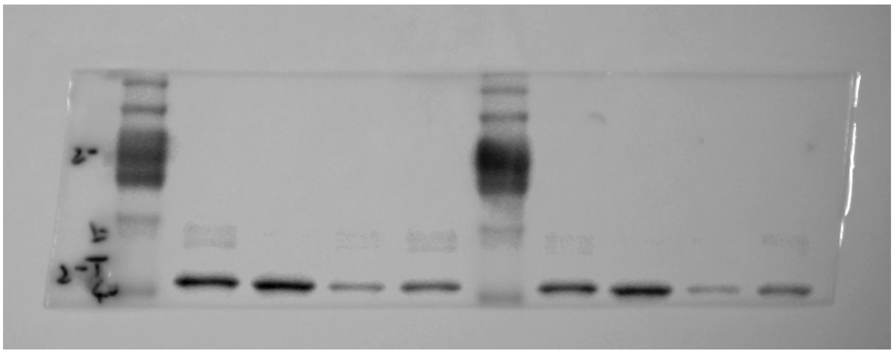

GOT1

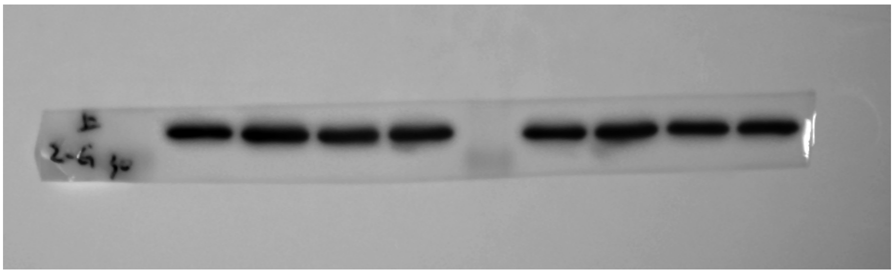

GAPDH

Marker WT NC Sh1-GOT Sh2-GOT Marker WT NC Sh1-GOT Sh2-GOT

TU686

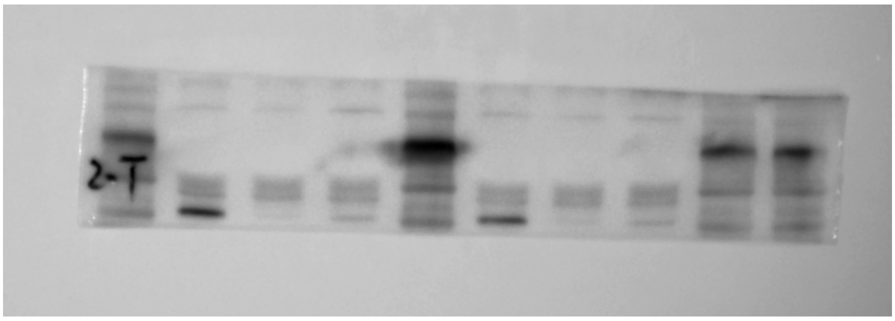

GOT1

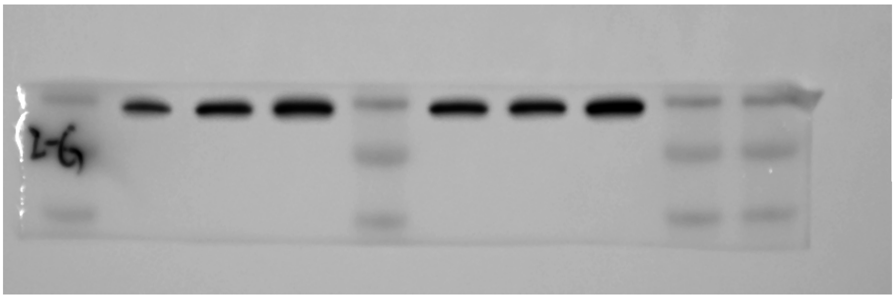

GAPDH

Marker NC Sh1-GOT Sh2-GOT Marker NC Sh1-GOT Sh2-GOT Marker Marker

Original western blot for Figure 6C

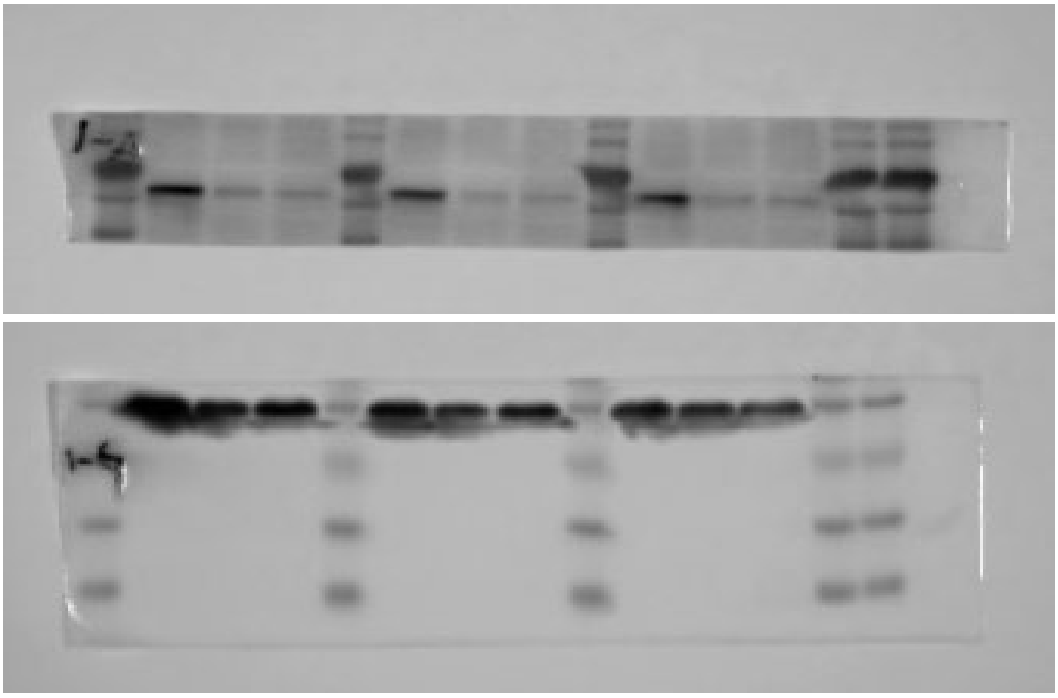

ALDH3A1

GAPDH

Marker NC Sh1 Sh2 Marker NC Sh1 Sh2 Marker NC Sh1 Sh2 Marker Marker

Original western blot for Figure 6D

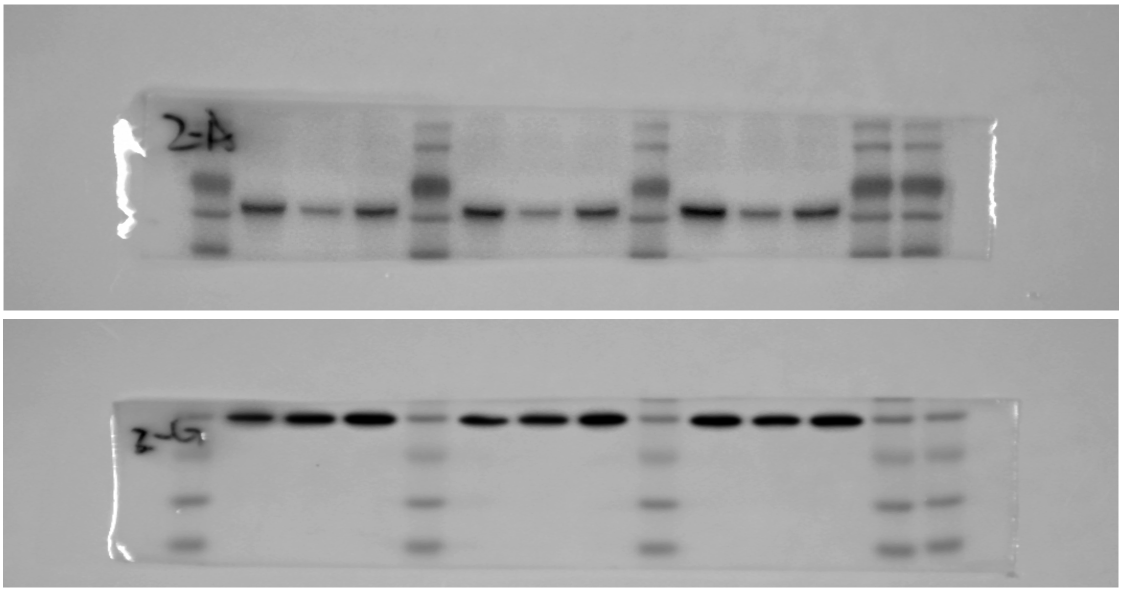

ALDH3A1

GAPDH

Marker NC Sh1 Sh2 Marker NC Sh1 Sh2 Marker NC Sh1 Sh2 Marker Marker
